# A Novel Framework for Qualification of a Composite-Based Main Landing Gear Strut of a Lightweight Aircraft

**DOI:** 10.3390/polym15061402

**Published:** 2023-03-11

**Authors:** Muhammad Ayaz Ahmad, Syed Irtiza Ali Shah, Sabih Ahmad Khan, Haris Ali Khan, Taimur Ali Shams

**Affiliations:** Department of Aerospace Engineering, College of Aeronautical Engineering, National University of Sciences and Technology, Islamabad 44000, Pakistan

**Keywords:** composite landing gear, design and analysis, qualification framework, testing and qualification methodology, CFRP manufacturing, drop test

## Abstract

The determination of suitable testing and qualification procedures for fiber-reinforced polymer matrix composite structures is an active area of research due to the increased demand, especially in the field of aerospace. This research illustrates the development of a generic qualification framework for a composite-based main landing gear strut of a lightweight aircraft. For this purpose, a landing gear strut composed of T700 carbon fiber/epoxy material was designed and analyzed for a given lightweight aircraft having mass of 1600 kg. Computational analysis was performed on ABAQUS CAE^®^ to evaluate the maximum stresses and critical failure modes encountered during one-point landing condition as defined in the UAV Systems Airworthiness Requirements (USAR) and Air Worthiness Standards FAA FAR Part 23. A three-step qualification framework including material, process and product-based qualification was then proposed against these maximum stresses and failure modes. The proposed framework revolves around the destructive testing of specimens initially as per ASTM standards D 7264 and D 2344, followed by defining the autoclave process parameters and customized testing of thick specimens to evaluate material strength against the maximum stresses in specific failure modes of the main landing gear strut. Once the desired strength of the specimens was achieved based on material and process qualifications, qualification criteria for the main landing gear strut were proposed which would not only serve as an alternative to drop test the landing gear struts as defined in air worthiness standards during mass production, but would also give confidence to manufacturers to undertake the manufacturing of main landing gear struts using qualified material and process parameters.

## 1. Introduction

Composite materials are extensively utilized in aerospace, marine, civil, automotive, and sporting applications [1] owing to their exceptional physical, mechanical, and thermal characteristics, especially their high stiffness and strength-to-weight ratios, superior fatigue strength, great corrosion resistance, and dimensional stabilities. The landing gear system is one of the most important systems in an aircraft’s as it withstand significant structural stresses that are encountered during landing. The landing process is the most critical phase in the flight operation as it involves a massive amount of energy transfer due to the landing impact, and the system of the aircraft is required to be stable enough so that it can operate successfully under these conditions [2]. Structural rigidity is one of the important design requirements for landing gear because it absorbs kinetic energy of the vertical load and it causes a reduction in the impact energy that causes vibrations at touchdown [3]. Most of the composite-based landing gear struts are non-retractable; however, designers of landing gear systems are now working on the development of retractable landing gear systems as well to improve the aircraft performance, payload capacity, and fuel efficiency [4]. A review of the literature suggests that new areas of research including numerical simulations, curing processes, and experimental testing of composite materials have been introduced and are also addressed in this research domain. Liviu Dragus et al. [5] designed a low-cost, low-mass landing gear system for an aerial target by performing the design calculation and analysis using a finite element approach. Using lamina macro mechanics and composite sheet resistance theories, simulations of the stresses in laminate composite materials are performed. After performing the computational analysis, experimental tests are conducted to validate the designed model.

**Figure 1:**
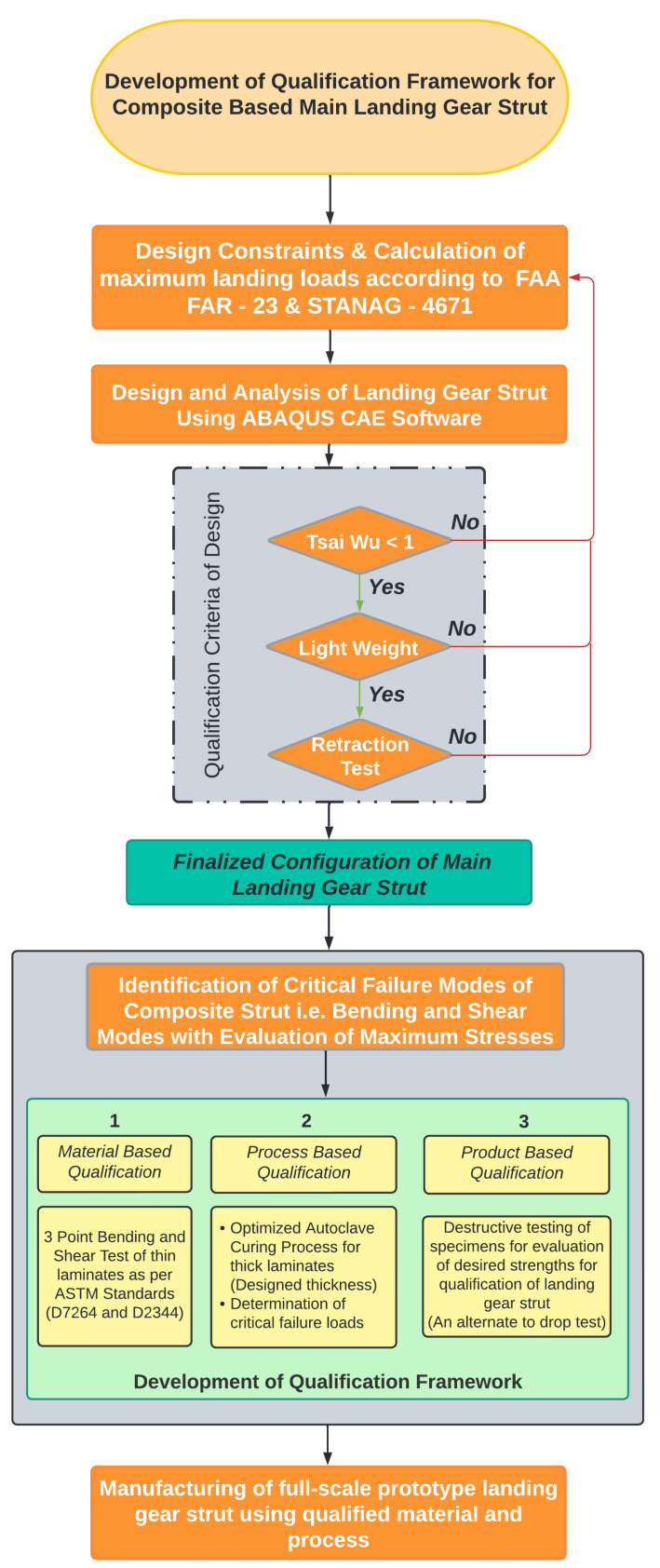
Proposed methodology for development of qualification framework for composite-based main landing gear strut.

Patunkar et al. [6] presented a comparison of a suspension system composed of glass fiber and steel leaf spring after carrying out their complete design and analysis using Pro-E^®^ and ANSYS 10.0^®^. The results presented in their research work showed that the deflection of the steel leaf spring was better than the composite leaf spring but at the cost of weight. A significant weight reduction of 84.4% was achieved by using a composite leaf spring suspension system. Xue et al. [7] also used a flexible leaf spring and presented a flexible multi-body dynamics model for the aircraft suspension system. Similarly, carbon-fiber-based landing gear struts were also presented by Liang et al. [8] with complete computational design and analysis and experimental validation. A comprehensive selection methodology for fiber-reinforced composite materials for retractable main landing gear struts of a lightweight aircraft up to 1600 kg mass is proposed by the authors in one of their recent studies [9]. In our previous work, four different fiber-reinforced composite materials were used for the design of the main landing gear struts of the aircraft under one-point landing condition. Finally, a material having a maximum strength-to-weight ratio and qualifying Tsai-Wu failure criterion was recommended for manufacturing the struts. Once the landing gear strut is manufactured based on the qualified design and using the selected material, a full-scale prototype is qualified for further installation on an aircraft subject to the clearance of a drop test as per the standard defined in the UAV Systems Airworthiness Requirements (USAR) [10] and Air Worthiness Standards FAA FAR Part 23 [11]. In compliance with the drop test, either a complete aircraft or a landing gear unit is used for the drop test to validate the strength of the landing gear system. For this purpose, the limits for drop test heights are between 9.2 inches and 18.7 inches as per the standards mentioned above. However, no qualification framework is available during mass production for conventional as well as composite-based landing gear struts. In the same context, the research aimed to propose the qualification criteria which would not only be required to be fulfilled before undertaking the full-scale manufacturing task of main landing gear struts but would also be used as the qualification criteria of the main landing gear struts of the given aircraft during mass production. As no studies are available in the literature that are relevant to qualification criteria of composite main landing gear struts during mass production, the methodologies proposed in this research are therefore considered to be novel, and are shown in Figure 1. This aim is achieved through developing a qualification criterion for the mass production by using available ASTM testing standards for the evaluation of mechanical properties of specimens. As bending and shear stresses are the most critical and prominent stresses encountered by the landing gear struts, bending and shear strength of the composite material can therefore be evaluated as per ASTM standards D 7264 [12] and D 2344 [13], respectively. Subsequently, the evaluation of these strengths can then be compared with the respective stresses and a logical framework can be developed for a qualification.

There are five sections in this research article. Section 2, which follows the Introduction, covers the design and analysis of a retractable landing gear strut for a given aircraft. Section 3 proposes a three-step qualification framework based on experimental techniques for material, process, and product. Required future work as a continuation of this research work is discussed in Section 4 followed by Conclusions in Section 5.

## 2. Details of the Proposed Methodology

### 2.1. Design Constraints

Table 1 depicts the constraints for the design and analysis of retractable main landing gear struts for which the qualification criterion has been developed in this research work.

The design constraints for this research work were provided by the aircraft’s original equipment manufacturer (OEM) and the same design constraints were followed during computational analysis in our previous research work [9] as well as for this study. Load factor means the ratio of a specified load to the total weight of the aircraft. For the design of the landing gear structure, a load factor of 2.667 is considered for small-category general aviation aircrafts according to NATO STANAG 4671 [10]. According to landing gear design principles present in the literature [14,15], and Airworthiness standards [10,11], the extreme landing condition is crash landing and the landing velocity must be 1.2 times the sink speed, and sink speed must be between 7 ft/s (2.13 m/s) and 10 ft/s (3.04 m/s). However, for the current study, the sink speed of the aircraft was 7.3 ft/s and hence the landing speed was kept at 1.2 times (2.667 m/s) for further consideration of the design and analysis of composite-based main landing gear strut and the same was provided by aircraft OEM.

### 2.2. Design and Analysis of Retractable Composite Main Landing Gear Strut

The selection methodology of composite materials for the design and development of main landing gear struts as proposed in our previous work [9] was followed to select the best available composite material with the maximum strength-to-weight ratio and qualifying Tsai-Wu failure criterion. Initial CAD models of the strut were developed within the given geometric constraints to ensure smooth retraction of the landing gear assembly inside the fuselage and to maintain the required ground clearance of the aircraft as well. Computational analysis using ABAQUS CAE^®^ was performed under maximum landing loads as per the specification mentioned in the UAV Systems Airworthiness Requirements (USAR) and Air Worthiness Standards FAA FAR Part 23 to identify the high-stress regions and critical failure modes encountered during landing by the main landing gear struts.

#### 2.2.1. Selection of Material

A unidirectional (UD) composite material composed of T300 carbon fiber/epoxy was recommended in our previous work for the manufacturing of the main landing gear strut for a lightweight aircraft of up to 1600 kg mass [9]. However, given the market availability of another unidirectional (UD) composite material composed of T700 carbon fiber/epoxy with improved mechanical properties, this one was additionally selected for analysis and subjected to the same analytical and computational analyses for comparison and qualification based on the Tsai-Wu failure criterion and strength-to-weight ratio. This repetitive work aimed to further improve and validate the desired characteristics of the intended product, based on the results obtained by using T700 carbon fiber/epoxy material. The properties of T700 carbon fiber/epoxy material are mentioned in Table 2. T300 carbon fiber and T700 carbon fiber are standard modulus carbon fibers and are well-recognized in the aerospace industry. From the performance point of view, carbon fiber T300 and T700 have the same tensile modulus of 230 GPa and 7μm diameter. However, a comparison of the mechanical properties of both carbon fibers shows a significant difference of 38.8% improved tensile strength of the T700 carbon fiber at the cost of only a 2.27% increase in volume density. The high tensile strength and flexibility of T700-based carbon fiber composite materials significantly reduce the probability of cracking and cracking of the produced carbon fiber products and hence their use has increased manifold in the aerospace-related industry (drones, UAVs, etc). They are mainly used to design frames or arms of drones and other structural members of UAVs that withstand impact loads as they are resistant to delamination on impact during flying. Based on our proposed methodology of material selection [9], the following steps were followed to evaluate the desired characteristics of the landing gear strut composed of T700 carbon fiber/epoxy material:

1. Calculation of mechanical properties using empirical methods.
2. Mechanical response of the thin laminated structure under axial loading conditions.
3. Mechanical behavior of thick composite beam under bending load.
4. Design and analysis of retractable main landing gear strut under one-point landing conditions.

A detailed analysis based on the steps mentioned above was performed for T700 carbon fiber/epoxy material to check the feasibility of the material for the manufacture of landing gear struts for the given aircraft. To avoid repetition of our previous research work, only the final results of the analysis are presented in the subsequent paragraphs.

#### 2.2.2. Design Finalization through Computational Analysis

**Figure 2:**
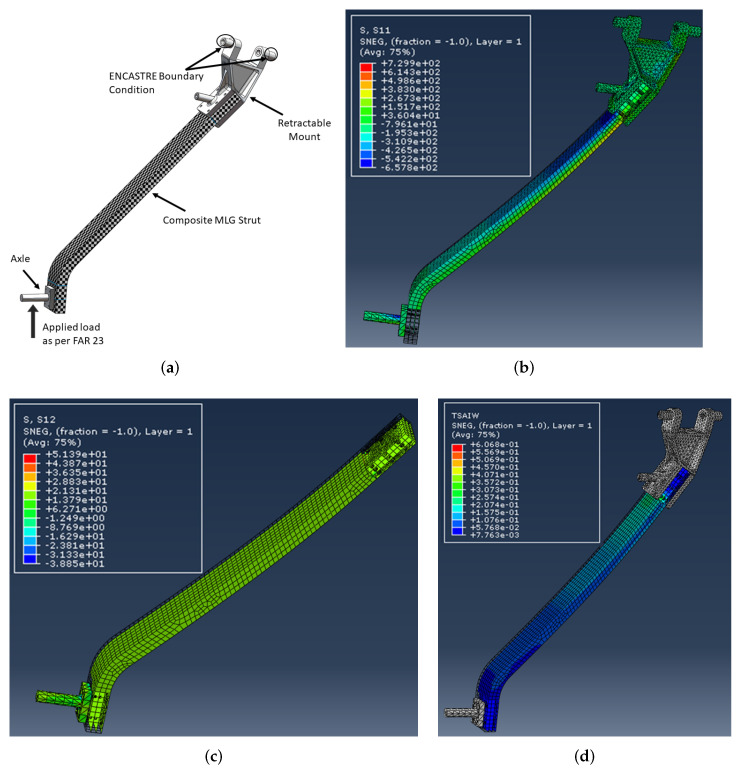
Design of the main landing gear strut and results of computational analysis under one-point landing condition: (**a**) Design of main landing gear assembly and applied boundary conditions. (**b**) Determination of maximum bending stress. (**c**) Determination of maximum shear stress. (**d**) Qualification of landing gear strut according to Tsai-Wu failure criterion.

SOLIDWORKS^®^ was used to develop CAD models of different dimensions of the main landing gear strut for the given aircraft within the given geometric constraints to ensure smooth retraction of the landing gear assembly inside the fuselage and to maintain the required ground clearance as well. After considering different combinations of dimensions and following the computational analysis for evaluating the designed landing gear struts against the Tsai-Wu failure criterion and strength-to-weight ratio, a design with constant width along the length was finalized. For computational analysis, the main landing gear strut was defined as a continuum shell and lay-up was performed in ABAQUS CAE^®^ composite lay-up module. The sub-assemblies were modeled as metal, and a 30CrMnSiA alloy material was assigned. The hybrid joint between the sub-assemblies and composite main landing gear strut was modeled with tie constraint interaction. Eight-node quadrilateral elements of type SC8R were used for modeling composite landing gear strut. However, the metallic sub assemblies were modeled with mesh element type C3D10. A total of 82,820 elements were utilized for computational analysis of the assembly. Figure 2a depicts the initial CAD model of the main landing gear assembly and applied boundary conditions that were analyzed using ABAQUS CAE^®^. The main landing gear assembly as depicted in Figure 2a is composed of a retractable mounting bracket, a composite strut, and an axle for tire assembly. The landing gear strut is fixed within the retractable mounting bracket and has ENCASTRE boundary condition. A vertical load of 41,865 N was applied to the landing gear strut from the axle to a simulate one-point landing condition. Figure 2a–d depicts the complete design of the main landing gear assembly, results of maximum bending and shear stresses, along with the Tsai-Wu failure criterion value of 0.61 encountered by the landing gear strut under given loads.

It is pertinent to mention that the main purpose of carrying out this computational analysis was not only to evaluate the results of the Tsai-Wu failure factor, mass and deflection of the strut made up of T700 carbon fiber/epoxy, but also to identify the localized stress concentration points and failure modes in the landing gear assembly. From the analysis, it is evident that bending and shear stresses dominate all kind of stresses and are localized around the bolt holes due to the high-stress concentration in landing gear assembly under given loads, with values of 729.29 MPa and 51.39 MPa, respectively, as depicted in Figure 2b,c. Thus, these would be considered predominant factors in defining and developing qualification criteria for composite landing gear struts during mass production.

Keeping in view the stress concentration around the bolt holes of the landing gear assembly with the installed mounting bracket and to improve the Tsai-Wu failure factor in an acceptable range under such conditions, the option of a hybrid joint with the reduced thickness-to-width ratio is preferred as already recommended in our previous research work. A comparison of T700 carbon fiber/epoxy and previously recommended T300 carbon fibre/epoxy material results shows improved performance of T700 carbon fiber/epoxy material in terms of Tsai-Wu failure factor value and mass of the strut. Details of the same are shown in Table 3.

## 3. Methodology for Qualification Framework

Once the design of a retractable main landing gear strut using T700 carbon fiber/epoxy was finalized, it was deemed essential to qualify the selected material, its curing process, and the required strength to overcome the bending and shear stresses encountered by the landing gear strut during one-point landing condition. In this context, a three-stepped qualification framework based on material, process, and product is formulated. Details of this three-stepped strategy for qualification of landing gear struts are (a) material-based qualification, (b) process-based qualification, and (c) product-based qualification, and details of the following are presented in subsequent sub-sections.

### 3.1. Material-Based Qualification

The bending and shear strength of the material were evaluated as per ASTM standards D 7264 and D 2344, respectively, for the qualification of material for main landing gear struts. At least five (05) specimens are required to be tested for a valid result as defined in the ASTM standards. However, to ensure maximum accuracy of results, a total of seven (7) specimens each were prepared for the bending and shear strength tests. A servo-controlled electric universal testing machine (UTM) was used to test these specimens. Dimensions of the test specimens were set as per the criteria defined in the ASTM standards and the specimens were cured in the autoclave following Manufacturer Recommended Curing Cycle (MRCC). For the bending strength evaluation of the material, the ratio of support length to thickness was maintained at 36:1 and the width of the sample was kept at 13 mm. Similarly, for shear strength evaluation of the material, the width of the specimen was taken as twice the thickness of the specimen. Results of the same are shown in Table 4 and Table 5.

According to the results obtained from these test specimens, the average bending and shear strengths of T700 carbon fiber/epoxy material were found to be 1211.42 MPa (SD = 61.4) and 70.5 MPa (SD = 4.9), respectively. Subsequently, these experimental results of bending and shear strength were compared with the maximum bending and shear stresses 729.29 MPa and 51.39 MPa encountered by the landing gear strut under one-point landing condition as calculated computationally using ABAQUS CAE^®^ and shown in Figure 2b,c, respectively. Based on this comparison between computational stresses and experimental mechanical properties, such as bending and shear strength, the selected unidirectional carbon-fiber-based composite pre-preg material T700 carbon fiber/epoxy was qualified on the material basis and was further recommended for process-based qualification against the designed thickness of the landing gear strut.

### 3.2. Process-Based Qualification

After the successful qualification of the T700 carbon fiber/epoxy material, the next step was to qualify the process for the thick laminate the of required thickness (38 mm) as recommended based on the computational analysis carried out for the complete landing gear strut under one-point landing condition using ABAQUS CAE^®^. It is also pertinent to mention that the thickness of the landing gear strut is kept constant throughout the length. It must be noted that the change in thickness may result in non-uniform curing during the autoclave process which may adversely affect the desired mechanical properties. Moreover, other manufacturing constraints, such as cutting, grinding, edge radius cutting, and post-autoclave machining, will also be associated with variation of thickness so the thickness will be kept constant throughout the length. This qualification step posed two major challenges including monitoring the gelation temperature at the mid-plane of full-thickness specimen and determination of applied pressure to avoid delamination due to the thickness of the laminate. Once the strategies to handle these two challenges were defined and validated through experimental testing, respective strengths were evaluated and compared with the required strengths against the computationally calculated stresses. In case of satisfactory results of this comparison, the second step of our qualification framework based on the process would then be declared qualified.

#### 3.2.1. Determination of Gelation Temperature at the Mid-Plane of Thick Specimen

**Figure 3:**
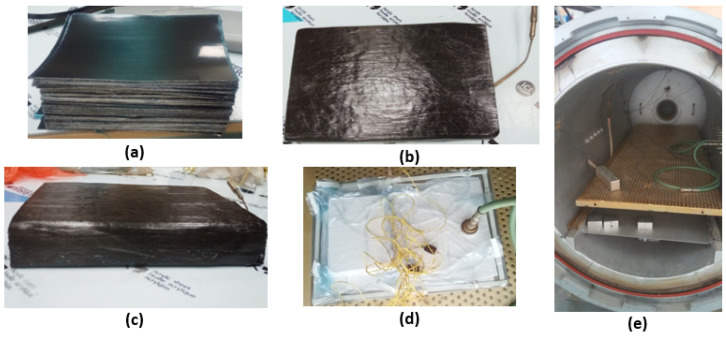
Defining of autoclave curing process for thick laminate: (**a**) Cutting of the pre-preg sheets on Zund cutter plotter. (**b**) Installation of the thermocouple at the mid-plane. (**c**) Final ply layup of required thickness. (**d**,**e**) Placement of uncured prepared laminate in the autoclave.

At first, pre-preg sheets of T700 carbon fiber/epoxy material were cut using Zund Cutter Plotter. A total of 300 pre-preg sheets each of 0.126 mm thickness were pre-processed through vacuum bagging. A ramp temperature of 2 ${}^{\circ }$C/min was defined in three stages for the autoclave process before the gelation temperature for pre-heating of the pre-preg sheets as mentioned in MRCC. To overcome the first challenge, a strategy was adopted and a thermocouple was installed at the mid-plane during the vacuum bagging of the specimens to monitor the temperature profile within the composite specimens to define the curing cycle of the material for the autoclave. It is pertinent to mention that as the curing process of pre-preg materials is an exothermic process, it is therefore required that the cycle for the autoclave is defined based on mid-plane temperature instead of uniform temperature within the autoclave for the sake of accuracy in terms of defining the curing process concerning temperature and time. Figure 3a–e show the complete processes from the cutting of pre-preg sheets to the curing of specimens in an autoclave.

#### 3.2.2. Determination of Applied Pressure for Autoclave Curing Process

**Figure 4:**
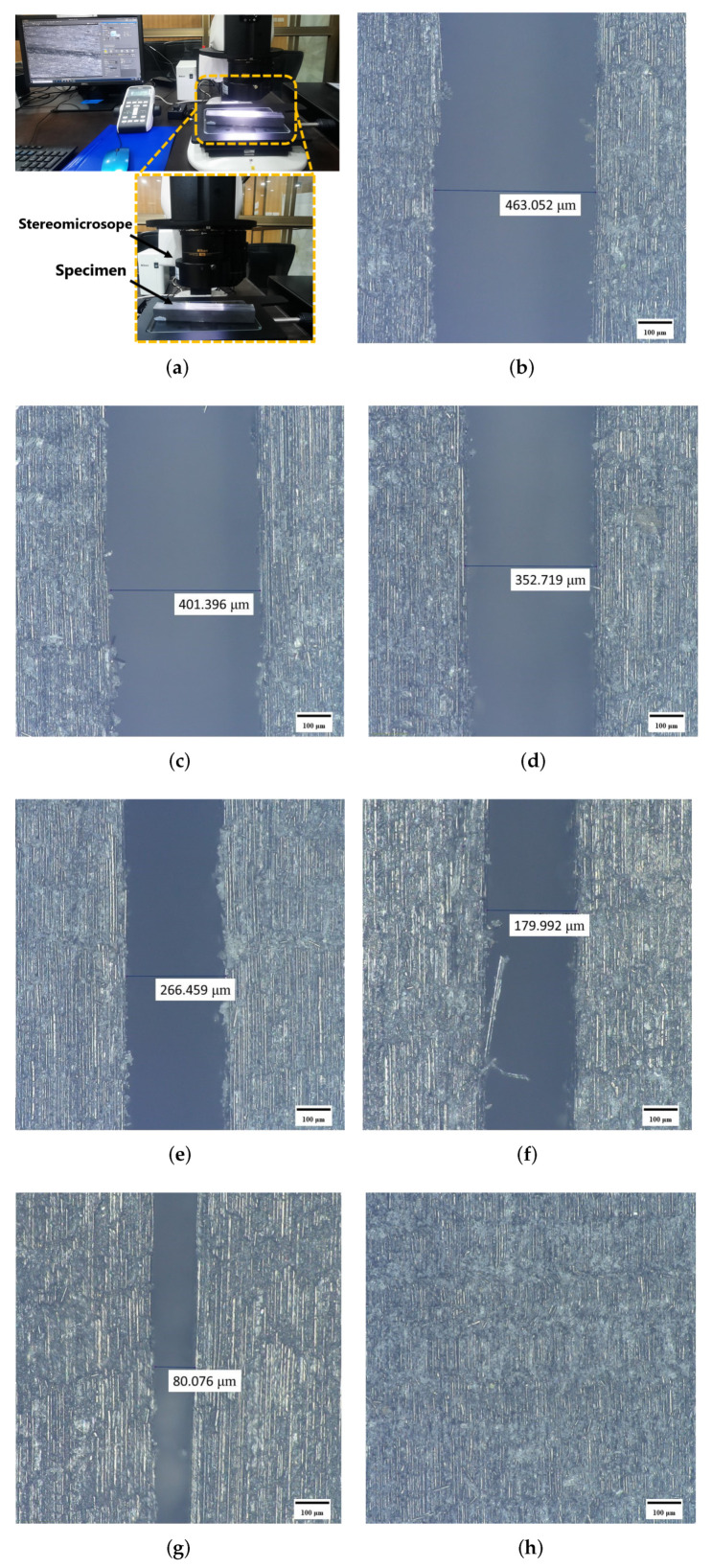
Stereo micrographs depicting the void reduction in the laminates under high pressure. (**a**) Setup for stereo microscopy. (**b**) A void of 463.052 μm was observed at 1 bar pressure. (**c**) A void of 401.396 μm was observed at 2 bar pressure. (**d**) A void of 352.719 μm was observed at 3 bar pressure. (**e**) A void of 266.459 μm was observed at 4 bar pressure. (**f**) A void of 179.992 μm was observed at 5 bar pressure. (**g**) A void of 80.076 μm was observed at 6 bar pressure. (**h**) No significant voids were observed at 7 bar pressure.

**Figure 5:**
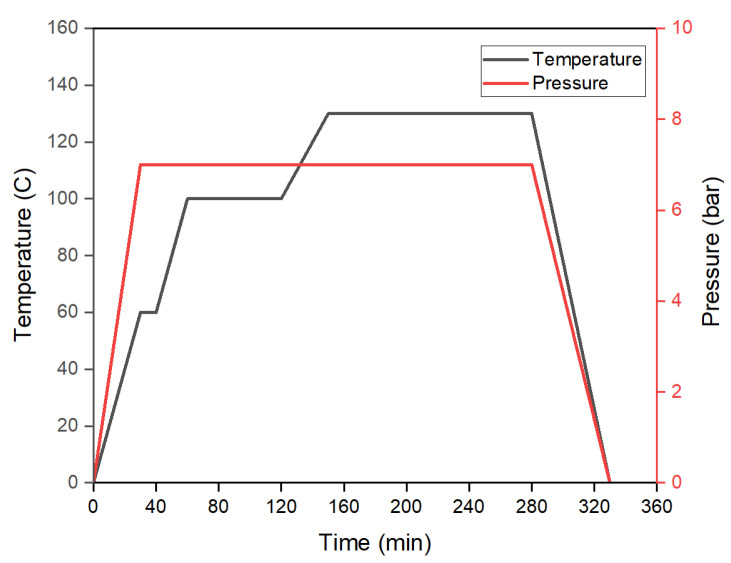
T700 carbon fiber/epoxy material curing cycle for 38 mm thick laminate.

It is worth mentioning that for the curing the thin specimen, only curing temperature and time are considered as the key parameters as provided in MRCC by the manufacturer. However, for the curing of thick laminates, the parameter of pressure also becomes equally important and thus determination of an optimum pressure requires experimental analysis to ensure proper curing of the pre-preg material. Moreover, it is worth mentioning that a very limited body of literature is available to address such technical concerns in the autoclave curing of thick pre-preg laminates. Hence, a strategy based on a non-destructive testing technique of cured laminates prepared at different applied pressures was developed. In this regard, specimens were cured using MRCC at different pressures within the autoclave starting from 1 bar vacuum and applied pressure each followed by an increment of 1 bar applied pressure in subsequent curing processes. A water-cooled diamond cutter was utilized to cut out the specimens from the cured thick blocks manufactured at different pressures and following the MRCC temperature profile. Cross-sections of the cured specimens were closely examined using a stereo microscope along the thickness to identify voids and delamination encountered during processing as shown in Figure 4a. The purpose of this analysis was to select the optimum pressure cycle for the curing of the laminate of the required thickness without significant delamination. A pictorial representation showing the characterization of specimens under a microscope for each specimen prepared at different curing parameters is shown in Figure 4b–h. It is evident from micrographs that the increase in pressure has a positive effect on the sample preparation thus minimizing the delamination of the laminae of the cured specimen from 463μm at 1 bar pressure to no significant voids at 7 bar pressure. The phenomena of void reduction under high pressure has also been discussed by [17,18,19]. Thus, the curing process was refined by increasing the applied pressure for the laminate of the required thickness and no significant delamination was observed for the specimen cured at 7 bar applied pressure. Hence, 7 bar applied pressure and 1 bar vacuum pressure along with the MRCC were recommended for the curing of thick laminate for subsequent manufacturing of the landing gear strut as shown in Figure 5.

#### 3.2.3. Determination of Critical Length for Evaluation of Bending and Shear Strengths

Once the curing cycle parameters as defined in MRCC and based on the above explained experimental works were defined, and specimens were prepared to validate the desired bending and shear strengths. ASTM standards D7264 and D 2344 can only be used for validation of mechanical properties of thin specimens as used in Section 3.1. However, no standard criterion is available for the validation of the mechanical properties of thick composite laminates. Therefore, an initial matrix was formed analytically using formulae written in the form of Equations (1) and (2) for the evaluation of bending and shear breaking forces, respectively, by using the bending and shear strengths calculated experimentally as defined in Section 3.1 above.

(1) $$\sigma_{b}=\frac{3\times F\times l}{2\times w\times t^{2}}$$

(2) $$\sigma_{s}=\frac{3\times F}{4\times w\times t}$$

**Figure 6:**
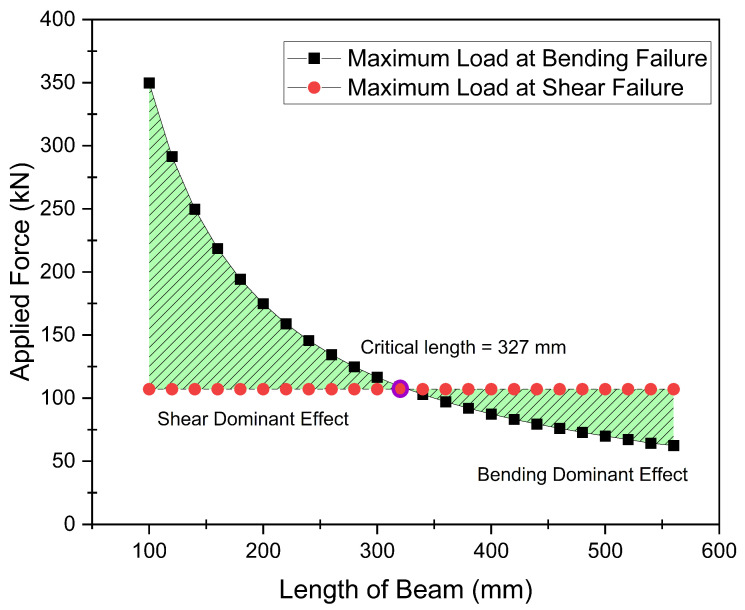
Determination of critical length for evaluation of bending and shear strengths.

It is pertinent to mention that shear force does not change with varying length of the specimen. However, the value of bending force is greatly affected by the length of the specimen as shown in Table 6. As the bending and shear strengths of the material were already calculated as 1211.42 MPa and 70.5 MPa, respectively, in Section 3.1 above, it was important to evaluate the bending and shear forces required against these strengths for different dimensions of the specimens. For this purpose, the thickness of the specimen was fixed as 38 mm based on the computational analysis carried out for the complete landing gear strut. Similarly, a constant width of 30 mm was used for all the specimens as both stresses are inversely proportional to the width of the specimen. Varying lengths of the specimens were taken, starting from 100 mm to 560 mm against the experimentally calculated bending and shear strengths of the material to evaluate the corresponding bending and shear forces. It is pertinent to note that these values of analytically calculated forces were required for comparison with the experimentally calculated forces to validate the actual bending and shear strengths of the material for thick laminates. Moreover, a graph was plotted as shown in Figure 6 to determine the critical length of the specimen beyond which the bending force dominated the shear force and the specimen would break under the bending stress before the shear stress. As a result, a specimen length of 327 mm was specified, beyond which failure would occur in bending mode.

**Figure 7:**
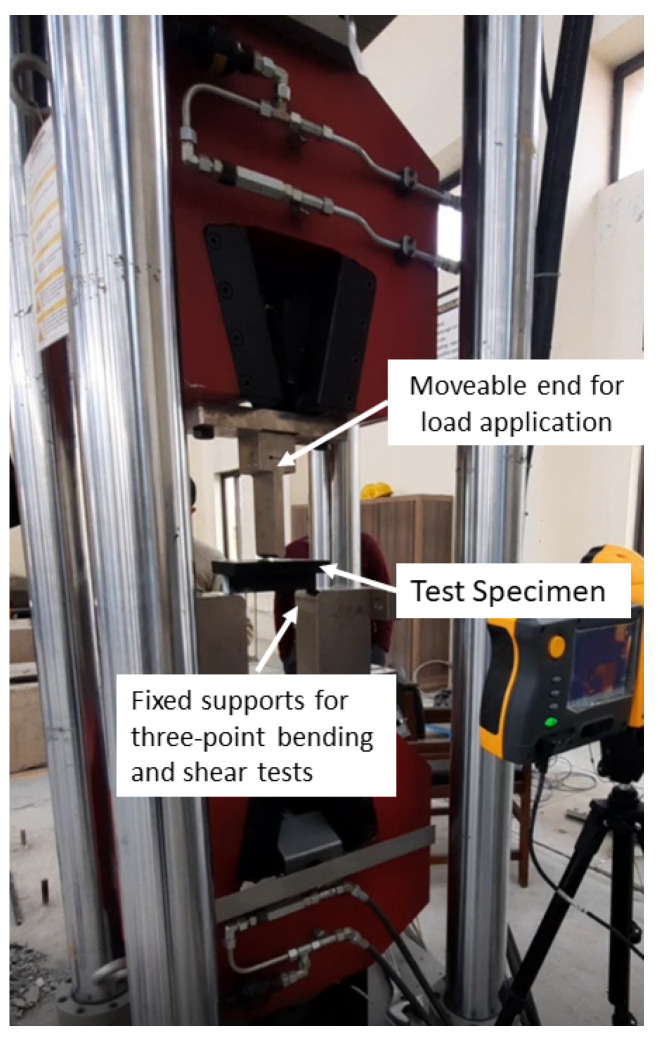
Three-point bending and shear test setup.

Subsequently, three samples each of three specimens above and below the critical length were taken and cured in an autoclave as per the defined curing cycle to obtain the experimentally calculated values of bending and shear forces. Figure 7 shows the complete experimental set-up used for this destructive testing. Results of the same destructive testing using the servo-controlled universal testing machine are shown in Table 7 and Table 8.

**Figure 8:**
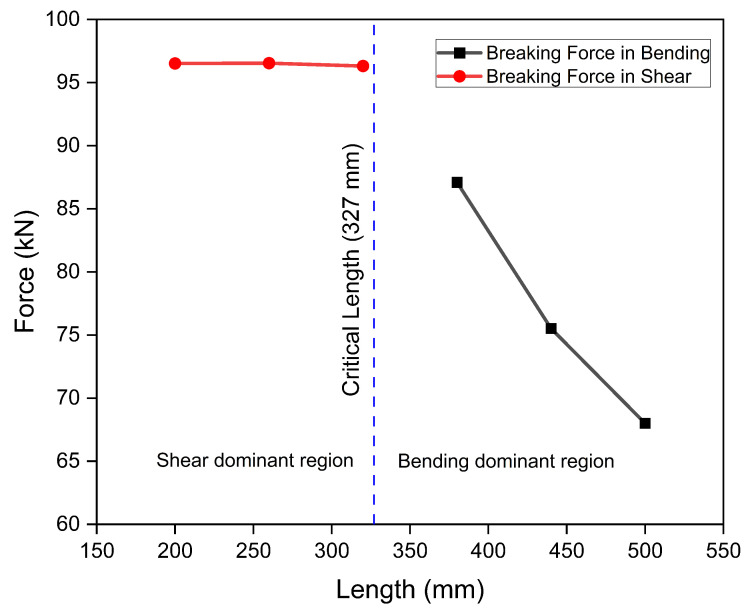
Effect of length on break force in shear and bending modes.

Based on this experimental analysis, average bending and shear strengths for the thick laminates against the designed thickness of 38 mm were found to be 1137.55 MPa and 63.33 MPa, respectively. On comparison with the experimentally calculated strengths for thin specimens, a 5–10 percent difference was observed for these experimentally calculated strengths of thick specimens mainly due to manufacturing/curing flaws. Once the average bending and shear strengths of thick specimens were defined, these were compared with the maximum bending and shear stresses encountered by the complete landing gear strut under a one-point landing condition. As the bending and shear strengths calculated based on experimental break force of the thick specimens as shown in Figure 8 were found to be 1137.55 MPa and 63.33 MPa, respectively, which were more than the maximum bending and shear stresses of 729.29 MPa and 51.39, respectively, encountered during one-point landing by the landing gear strut, the process was therefore also qualified for the required thickness and recommended for further development of complete prototype strut. It is evident from Figure 8 that the break force in shear failure mode remains constant with varying length, however, the break force in bending failure decreases with an increase in length.

#### 3.2.4. Determination of Dimensions of Test Specimen

As these results obtained in the previous section were based on three different dimensions of the specimens by keeping the critical length as a central point, it was therefore deemed essential to define the dimensions of the test specimen for future work. In the same context, the length of shear and bending specimens would be kept 50 percent lower and greater than the critical length, respectively, to ensure the failure of the specimens in pure shear and bending modes under destructive testing. However, the thickness of the specimens would be kept equal to the actual thickness of the landing gear strut as required for process-based qualification with a constant width of 30 mm. The final dimensions of the test specimens for bending and shear strengths are tabulated in Table 9 and Table 10 below:

#### 3.2.5. Validation of Experimental Testing

**Figure 9:**
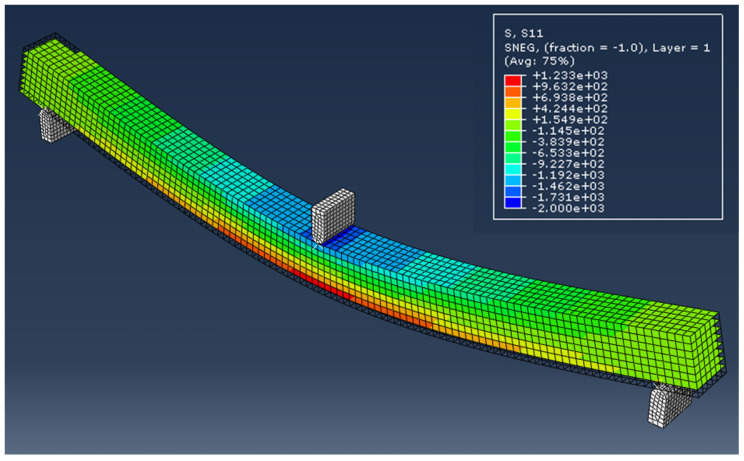
Three-point bending analysis of the specimen.

**Figure 10:**
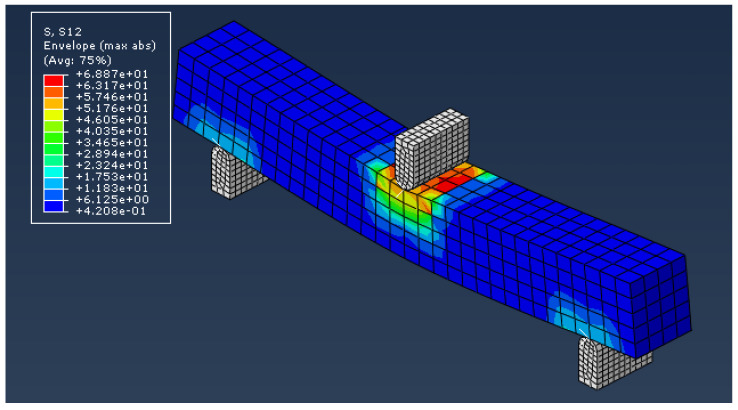
Three-point shear analysis of the specimen.

One of the experimental results of the T700 carbon fiber/epoxy material specimen was also compared with the computational analysis performed for bending and shear strength of the material using Abaqus CAE^®^. In this analysis, failure under bending and shear modes were closely examined and failure loads were compared with the experimental values obtained through destructive testing of the specimens. Figure 9 and Figure 10 show the results of these computational analyses. A comparison of these computational and experimental analyses depicts a variation of only 6–8%, which proves proximity in both approaches. Moreover, the computational model using Abaqus CAE^®^ adopted for the evaluation of maximum bending and shear loads under one-point landing condition for the main landing gear strut were also validated based on this comparison.

### 3.3. Product-Based Qualification

Considering the landing gear strut as a final product, it is pertinent to mention that the prototypes of all types of landing gear struts need to be qualified through drop test as specified in the UAV Systems Airworthiness Requirements (USAR) and Air Worthiness Standards FAA FAR Part 23. In compliance with the drop test, either a complete aircraft or a landing gear unit must be used for the drop test to validate the strength of the landing gear system. For this purpose, the drop height must not be less than the height calculated by Equation (3) [11]. However, the limits for drop test heights are between 9.2 inches and 18.7 inches.

(3) $$h=3.6\sqrt{\frac{W}{S}}$$

**Figure 11:**
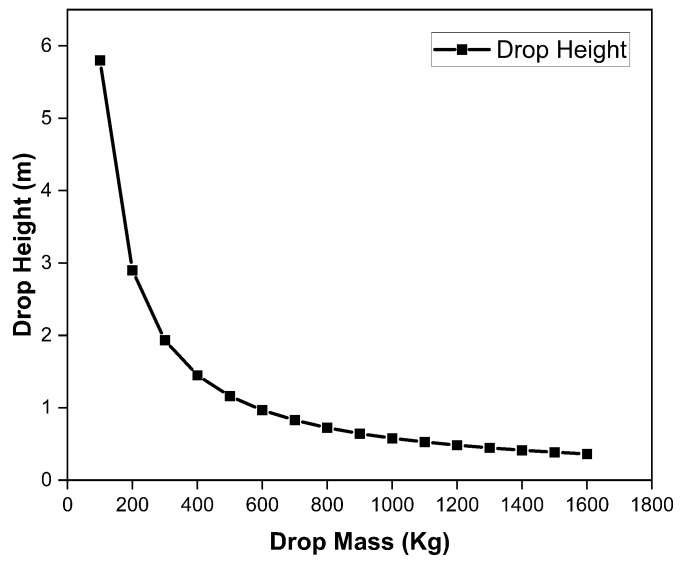
Variation of drop height with dropping mass for product-based qualification of landing gear strut.

A similar relation is also developed by equating the kinetic and potential energy of the system. Assuming a lift-to-weight ratio equal to 1, Equation (4) [20] can also be used for the calculation of drop height and its variation with the mass of the system as shown in Figure 11.

(4) $$H=\frac{1}{2g}\frac{m_{UAV}}{m_{lg}}V^{2}$$

However, this product qualification method only applies to the full-scale prototype during the development phase. No studies are available in the literature for the qualification of landing gear struts during mass production after drop test qualification. In the case of composite struts, these criteria become even more complex keeping in view the sensitivity of material properties and process limitations. Therefore, a comprehensive three-step qualification method is proposed in this research work. After material- and process-based qualification as discussed in detail above, an alternate qualification criterion to the drop test for the final product is also recommended. As the prototype of the landing gear strut has not been developed, the product would therefore be declared qualified by destructive testing of the specimens cured with the complete landing gear strut in the same autoclave cycle to validate the required strengths in bending and shear modes. In this context, maximum stresses encountered by the landing gear strut under one-point landing condition in bending and shear modes as calculated by computational analysis have been taken as the main design requirements of the final product. As destructive testing of the specimens provides the break force, lower limits of experimentally obtained break forces, therefore, need to be evaluated against the maximum bending and shear stresses with values of 729.29 MPa and 51.39 MPa, respectively, against the finalized dimensions of specimens as mentioned in Table 9 and Table 10. These lower limits of break forces are then used for the evaluation of the bending and shear strengths of the cured specimens of specified dimensions. The complete landing gear strut can then be declared qualified if the strengths of the specimens in bending and shear modes are found to be greater than the maximum bending and shear stresses of 729.90 MPa and 51.39 MPa, respectively.

## 4. Future Work

**Figure 12:**
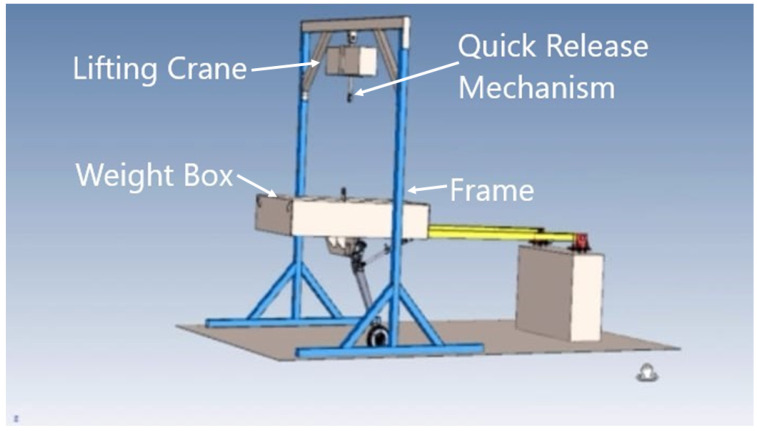
Conceptual design of drop test rig for product-based qualification of main landing gear struts of the given aircraft.

Based on the satisfactory results of the three-step qualification framework as proposed in this research work, manufacturing tasks of both of the composite main landing gear struts are recommended using T700 carbon fiber/epoxy material. The same autoclave cycle as defined in Section 3.2 would be followed to ensure proper curing of the thick laminated landing gear strut. Once full-scale prototype landing gear struts are developed, they would be required to undergo drop tests first as per the requirements of UAV Systems Airworthiness Requirements (USAR) and Air Worthiness Standards FAA FAR Part 23 to re-validate the proposed product-based qualification for mass production of the landing gear struts. A conceptual design of a drop test rig has also been proposed for the given aircraft which is designed to withstand a mass of 1600 kg with an adjustable maximum drop height of 1.8 m. It is also equipped with an electric lifting crane and a quick-release mechanism to ensure the free-fall motion of the weight box and as shown in Figure 12.

## 5. Conclusions

The major conclusion of the research work are as follows:

1. A novel framework for the qualification of a composite-based main landing gear strut of a lightweight aircraft has been formulated based on material, process, and product qualification.
2. The qualification framework is focused on enhancing the load-carrying capability of the strut in terms of bending and shear loads which are the key loads for a uniform cross-section beam.
3. The framework evaluates the load-carrying capability using a systematic two-pronged approach by leveraging computational and experimental testing for the selection of appropriate material and process parameters.
4. The proposed framework, which is based on sample/coupon testing, can be utilized for the full-scale prototyping of a main landing gear strut.

## Figures and Tables

**Table 1:** Design constraints for the main landing gear struts of the given aircraft.

Parameters	Values
Mass of the aircraft	1600 kg
Type of landing gear system	Composite leaf spring
Load factor	2.667
Landing velocity	2.67 m/s
Landing conditions	Crash landing

**Table 2:** Material properties of T700 carbon fiber/epoxy (Data acquired from [16]).

Parameters	Experimental Values
Density (kg/m^3^)	1570
Young’s modulus in X	132 GPa
Young’s modulus in Y	10.3 GPa
Young’s modulus in Z	10.3 GPa
Tensile strength in X	2100 MPa
Tensile strength in Y	24 MPa
Tensile strength in Z	65 MPa
Shear strength in X	75 MPa
Shear strength in Y	75 MPa
Shear strength in Z	75 MPa

**Table 3:** Dimensional and properties-based comparison of landing gear assemblies composed of T700 carbon fiber/epoxy and T300 carbon fiber/epoxy under one-point landing condition.

Material (UD Lamina)	Fiber Volume Ratio $\left(V_{f}\right)$	Thickness of Each Lamina	No. of Laminae/Piles	Thickness of the Strut (mm)	Width of Strut (mm)	Tsai-Wu Failure Criteria	Mass (kg)	Deflection (cm)
T300 carbon fiber/epoxy	0.70	0.24	220	52.8	100	0.8	10	19.60
T700 carbon fiber/epoxy	0.67	0.126	300	38	120	0.6	8	19.28

**Table 4:** Bending strength evaluation of T700 carbon fiber/epoxy material.

Sample No	Width of the Sample (m)	Thickness of the Sample (m)	Length of the Sample (m)	Breaking Force (N)	Bending Strength (MPa)
1	0.01289	0.00264	0.084	896.8	1265
2	0.01286	0.00269	0.086	882.9	1225
3	0.01291	0.00271	0.087	860.1	1180
4	0.01297	0.00268	0.086	905.2	1250
5	0.01286	0.00287	0.092	838.1	1090
6	0.01282	0.00265	0.085	891.8	1260
7	0.01289	0.00264	0.084	857.8	1210
Avg Values	0.01288	0.00269	0.086	876.1	1211.42

**Table 5:** Shear strength evaluation of T700 carbon fiber/epoxy material.

Sample No	Width of the Sample (m)	Thickness of the Sample (m)	Breaking Force (N)	Shear Strength (MPa)
1	0.00796	0.00398	2890.13	68.42
2	0.00754	0.00377	2733.05	72.11
3	0.00752	0.00376	2597.17	68.89
4	0.00744	0.00372	2648.12	71.76
5	0.008	0.004	3289.17	77.09
6	0.0075	0.00375	2306.63	61.51
7	0.00758	0.00379	2826.09	73.78
Avg Values	0.0076	0.00382	2755.76	70.50

**Table 6:** Analytical evaluation of bending and shear forces for varying lengths of the specimens to determine the critical length.

Specimen No	Length (m)	Width (m)	Thickness (m)	Bending Force (N)	Shear Force (N)
S-1	0.100	0.030	0.038	349,736.80	107,160
S-2	0.120	0.030	0.038	291,447.33	107,160
S-3	0.140	0.030	0.038	249,812.00	107,160
S-4	0.160	0.030	0.038	218,585.50	107,160
S-5	0.180	0.030	0.038	194,298.22	107160,
S-6	0.200	0.030	0.038	174,868.40	107,160
S-7	0.220	0.030	0.038	158,971.27	107,160
S-8	0.240	0.030	0.038	145,723.67	107,160
S-9	0.260	0.030	0.038	134,514.15	107,160
S-10	0.280	0.030	0.038	124,906.00	107,160
S-11	0.300	0.030	0.038	116,578.93	107,160
S-12	0.320	0.030	0.038	109,292.75	107,160
S-13	0.340	0.030	0.038	102,863.76	107,160
S-14	0.360	0.030	0.038	97,149.11	107,160
S-15	0.380	0.030	0.038	92,036.00	107,160
S-16	0.400	0.030	0.038	87,434.20	107,160
S-17	0.420	0.030	0.038	83,270.67	107,160
S-18	0.440	0.030	0.038	79,485.64	107,160
S-19	0.460	0.030	0.038	76,029.74	107,160
S-20	0.480	0.030	0.038	72,861.83	107,160
S-21	0.500	0.030	0.038	69,947.36	107,160
S-22	0.520	0.030	0.038	67,257.08	107,160
S-23	0.540	0.030	0.038	64,766.07	107,160
S-24	0.560	0.030	0.038	62,453.00	107,160

**Table 7:** Experimental evaluation of bending strength of the thick laminate.

Specimen No	Length (m)	Width (m)	Thickness (m)	Destructive Bending Force (N)	Bending Strength (MPa)
S-1	0.380	0.030	0.038	84,892	1117
S-2	0.380	0.030	0.038	87,476	1151
S-3	0.380	0.030	0.038	86,032	1132
Avg Values	0.380	0.030	0.038	86,133.33	1133.33
S-4	0.440	0.030	0.038	73,512	1120
S-5	0.440	0.030	0.038	75,744	1154
S-6	0.440	0.030	0.038	74,497	1141
Avg Values	0.440	0.030	0.038	74,584.33	1138.33
S-7	0.500	0.030	0.038	64,171	1111
S-8	0.500	0.030	0.038	67,982	1177
S-9	0.500	0.030	0.038	65,557	1135
Avg Values	0.500	0.030	0.038	65,903.33	1141.00

**Table 8:** Experimental evaluation of shear strength of thick laminate.

Specimen No	Length (m)	Width (m)	Thickness (m)	Destructive Shear Force (N)	Shear Strength (MPa)
S-1	0.200	0.030	0.038	96,520	63.5
S-2	0.200	0.030	0.038	95,304	62.7
S-3	0.200	0.030	0.038	96,216	63.3
Avg Values	0.200	0.030	0.038	96,013	63.16
S-4	0.260	0.030	0.038	95,608	62.9
S-5	0.260	0.030	0.038	97,097	63.88
S-6	0.260	0.030	0.038	96,504	63.49
Avg Values	0.260	0.030	0.038	96,403	63.42
S-7	0.320	0.030	0.038	95,912	63.1
S-8	0.320	0.030	0.038	96,976	63.8
S-9	0.320	0.030	0.038	96,337	63.38
Avg Values	0.320	0.030	0.038	96,408	63.42

**Table 9:** Dimensions of the test specimen for verification of bending strength.

Length (m)	Width (m)	Thickness (m)
0.500	0.030	0.038

**Table 10:** Dimensions of the test specimen for verification of shear strength.

Length (m)	Width (m)	Thickness (m)
0.200	0.030	0.038

## Data Availability

Not applicable.
